# Leveraging technologies for data management and sharing to foster collaboration and implement data spaces

**DOI:** 10.1016/j.dib.2026.112769

**Published:** 2026-04-17

**Authors:** Ioannis Chrysakis, Gerasimos Antzoulatos, Luis Sánchez, Josiane Xavier Parreira, Dimitrios Skoutas, Iroshani Jayawardene, Roberto Di Bernardo, Eloisa Vargiu, Stefanos Vrochidis, Erik Mannens

**Affiliations:** a Netcompany S.A.*,* Research and Innovation Development Department (RID)*,* Luxembourg; b IDLab*,* Department of Electronics and Information Systems*,* Ghent University*,* imec*,* Belgium; c DTAI*,* Department of Computer Science*,* KU Leuven*,* Belgium; d Centre For Research and Technology - Hellas, Greece; e Network Planning and Mobile Communications Lab*,* Universidad de Cantabria*,* Spain; f Siemens AG*,* Austria; g Athena Research Center*,* Greece; h Sintef Digital*,* Norway; i Engineering Group*,* Italy; j Cetaqua*,* Spain

**Keywords:** Data spaces, Data management, Data ecosystems, Data sharing

## Abstract

The 2020 European Strategy for Data aims at developing Common European Data Spaces as a means to build a pan-European single market for data, thereby supporting economic growth and maximizing citizens' use of data. It demands data spaces in strategic sectors, with capabilities for effective data management and sharing. Although several initiatives support their adoption, data spaces are still in the early stages of development and face several data management and sharing challenges. To identify the requirements needed to address these challenges, we review the literature in developing a conceptual framework for applying data management and sharing in the context of data spaces. We then evaluate the practical implementation of the proposed solutions by analysing six representative European-funded projects. Focusing on requirements from trust and business models to interoperability, workflow orchestration, energy efficiency, and data quality, the work highlights prominent issues and explains how each project addresses them through technical means. Our evaluation outlines each project's aim and contribution, along with a representative use case from different domains, e.g., water, agriculture, and energy. We recognize widely accepted strategies such as the use of semantic standards, data catalogues, distributed ledger technologies for trust enhancement, and federated identity management. This work highlights recurring patterns, common practices, and key differences in implementation and identifies open research gaps. Thus, it aims to inform future initiatives and provide concrete recommendations to researchers and practitioners on achieving data spaces with best practices. As the field matures, the work hopes to help achieve scalable, stable, and cross-domain data spaces that support sustainable innovation and long-term collaboration throughout Europe.

## 1 Introduction

The Common European Data Spaces initiative aims to enable a trustworthy, interoperable environment in which organisations can share and recirculate data securely, governed by pre-agreed guidelines, to ensure economic development, innovation, and openness [1,2]. Health, manufacturing, agriculture, mobility, and energy represent just a few examples of the Common European Data Spaces that will be created as part of the 2020 European Strategy for Data, published under the previous Commission (2019–2024) [3]. This indicates the strategic relevance of data sharing infrastructures for specific sectors in supporting Europe's digital transformation.

But the effort to implement data spaces as a means toward the European single market for data also brings considerable data management and sharing challenges, such as ensuring data trust, interoperability, and data quality [4,5]. From these challenges, a set of corresponding requirements has been formulated in policy and research literature, defining the technical and organisational conditions necessary for effective data-space implementation. Throughout this paper, the term “data spaces“ refers specifically to Common European Data Spaces, as defined in the 2020 European Strategy for Data [3]. Several European initiatives, e.g., IDSA [6] and GAIA-X [7], have been initiated to address these underlying challenges and the resulting requirements, by providing technical recommendations, software building blocks, reference architectures, and specifications for the implementation of data spaces. Another initiative of a different nature is the Data Spaces Support Centre (DSSC) [8]. While IDSA, GAIA-X, the FIWARE Foundation [9] and BDVA [10] are community-driven associations, the DSSC is a European Commission-funded initiative established to coordinate and align existing efforts toward a common blueprint for data spaces. The DSSC builds upon and integrates the contributions of of the aforementioned key reference organizations, each of which has developed relevant standards, architectures and specifications for data-space implementation. Indeed, the DSSC plays a critical role in the current implementation of data spaces. By implementing a minimum consensus architecture, the DSSC Blueprint [11] aims to reconcile disparate initiatives and provide a reusable framework for instantiating sectoral and cross-sectoral data spaces.

Current data space solutions, according to the future architectural vision outlined in the DSSC Blueprint, are increasingly being used by EU-funded projects, particularly under programmes such as Horizon Europe. The vast majority of them tackle data management and sharing challenges by implementing solutions that fulfil the requirements derived from those challenges, such as interoperability, workflow orchestration, metadata and quality management, data discovery, energy efficiency, trust, and business model design, tuning their approach to the specific needs of sectors such as water, energy, and agriculture. These efforts are in line with the goals of the European Green Deal, which is to establish robust and sustainable ecosystems [12].

As data space technologies continue to emerge and evolve rapidly, a detailed examination of their current deployment offers timely insights and strategic advantages for both researchers and practitioners. To illustrate the technological approaches adopted in the implementation of data spaces, the present paper provides a selective evaluation of six representative Horizon Europe projects funded under the call *HORIZON—CL4–2021-DATA-01–03: Technologies for data management* [13]. The study is guided by the following central research question:

*How can current EU-funded projects inform the technological foundations and best practices needed for implementing data spaces?*

This study contributes to the broader discussion on data spaces by offering an evidence-based comparative synthesis that supports the design of interoperable, secure, and sustainable data-sharing ecosystems, while deepening the understanding of enabling technologies, architectural options, and emerging best practices.

This paper presents four contributions discussing data space implementation. First, we review key challenges in data sharing and management and derive the corresponding requirements. Second, we explain the rationale behind the selection of the projects, highlight their key features, and outline their technical focus, which sets the stage for a qualitative cross-case analysis based on their publicly available outputs (SubSection 2.2). Third, we present a conceptual framework for data management and sharing (SubSection 2.3) within the context of data spaces, aligned with the three pillars defined under the DSSC Technical Building Blocks, namely Data Interoperability, Data Sovereignty and Trust, and Data Value Creation Enablers. This framework serves as the basis for our analysis. Building on this, in Section 3 we outline the offerings of each project along with a use case. In Section 4 we conduct a cross-project analysis to identify commonalities and differences in implementation, as well as open issues for future research to the evolving European Data Spaces landscape. Finally, Section 5 provides a summary and conclusions.

## 2 Methodology

In this section, we outline how we approach our research question. We first review key challenges reported in European policy documents and scientific literature, particularly in the European Strategy for Data [5] and related studies [14,15,16]. These challenges include limited interoperability, lack of trusted data-sharing mechanisms, heterogeneous data quality, fragmented discovery capabilities, energy-inefficient processing, and the absence of viable business and governance models. Based on these challenges, we derive a set of corresponding requirements that define the technical and organisational conditions necessary for effective data-space implementation. We then select European projects funded under the call *HORIZON—CL4–2021-DATA-01–03* that implement technological solutions to satisfy these requirements and thereby address the underlying data management and sharing challenges within the context of data spaces. To enable a structured cross-comparison, we introduce a conceptual framework that maps the identified requirements to the technological building blocks defined by the DSSC. This mapping facilitates analysis of how projects operationalize enablers such as interoperability and trust mechanisms to overcome shared obstacles and advance the implementation of data spaces.

### 2.1 Requirements for data management and sharing

Efficient data space implementation depends on meeting a set of interdisciplinary and interrelated requirements, which have been derived from persistent challenges in data sharing and management. These challenges, widely discussed in the literature and EU policy studies [5,[14], [15], [16]], highlight the need for clear technical and organizational conditions. Drawing on an analysis of the European Strategy for Data [3], the DSSC Blueprint V2.0 [11], and the relevant scientific literature (see Table 1), we identify seven overarching requirements prerequisite to efficient data management and sharing in data spaces: *interoperability, workflow orchestration, data quality, data discovery, energy efficiency, trust frameworks, and business models.*

**Table 1:** Data management and sharing requirements: extraction based on key references.

Requirement	Description	Key References
**Interoperability**	Enabling seamless data exchange across diverse systems via technical, semantic, and legal layers.	[3,11,[17], [18], [19], [20], [21]]
**Workflow Orchestration**	Automating and managing distributed data pipelines to enable scalable and reliable data processing.	[[13], [22], [23], [24], [25]]
**Data Quality**	Ensuring accuracy, completeness, consistency, and validity of data.	[[5], [26], [27], [28], [29], [30]]
**Data Discovery**	Facilitating the findability of datasets and services via rich metadata, catalogues, and search tools.	[2,4,11,[31], [32], [33]]
**Energy Efficiency**	Minimizing the environmental impact of data processing and calculating energy consumption, e.g., of Machine Learning (ML) Models.	[[34], [35], [36], [37], [38]]
**Trust Framework**	Establishing identity, access control, usage policies, and data sovereignty across federated systems.	[1,2,11,[39], [40], [41]]
**Business Models**	Designing value-sharing models and marketplaces to incentivise data sharing and reuse.	[[1], [2], [3],^11^,[42], [43], [44]]

In particular, our analysis is consistent with the classification proposed by the JRC report on European Data Spaces [16], which distinguishes between functional and non-functional requirements. In this report, the requirements were derived directly from European policy documents on data spaces, which justifies their inclusion in our analysis. Six of the identified requirements correspond to non-functional requirements in the JRC classification: interoperability relates to interoperability and standardisation; workflow orchestration aligns with performance and scalability as part of operational efficiency; data quality corresponds to quality and reliability; energy efficiency reflects sustainability and resource efficiency; trust frameworks align with trust, security, and compliance; and business models correspond to economic sustainability and value creation mechanisms. The remaining item, data discovery, corresponds to a functional requirement, namely data publication and discovery.

*Interoperability* is defined as the ability to enable seamless data sharing between heterogeneous systems [17] and is one of the core FAIR principles [31]. It is a core requirement for data management and sharing in data spaces, as it directly affects the capacity of diverse systems and organizations to understand, access, exchange, and use data in a meaningful and trustworthy way [18,[19], [20], [21]].

*Workflow orchestration* is essential for automating and managing distributed data pipelines [22]. In data spaces, it presents a key requirement, as it must coordinate complex, scalable, and trustworthy data processing across heterogeneous environments while ensuring quality, transparency, automation, and cross-organizational collaboration [[22], [23], [24], [25]^25^].

*Data quality* is fundamental for deriving accurate and dependable insights worthwhile for data sharing, largely determining the value of shared data within data spaces [26]. High data quality is essential for supporting productive analysis, informed decision-making, and operational efficiency within data spaces, where data is shared among stakeholders and sources [21,[27], [28], [29], [30]]. Poor data quality, inconsistent formats, or erroneous information undermines the reliability of analyses and reduces user trust. Over time, this discourages stakeholders from contributing and reusing data, thereby threatening the long-term sustainability of data ecosystems [5].

*Data discovery* is the process of finding, identifying, and comprehending datasets that are pertinent to a given task or use case*.* The volume and heterogeneity of data, the inadequate metadata currently in use, the high expense of manual curation, and the shortcomings of the search and preview tools make data discovery in data spaces a difficult task [2,4,11,32]. To facilitate efficient data sharing, it is essential to adopt FAIR principles in the process of data discovery [31,33].

*Energy efficiency* poses a major challenge in data spaces due to the large-scale, distributed, and heterogeneous nature of data management and sharing [34,35]. Energy consumption in transfer, processing, and storage increases when handling large amounts of heterogeneous data across different nodes (e.g., in data spaces). Although techniques like federated learning, edge computing, and data minimization can lower energy consumption, their lack of standardised energy metrics and their technical complexity hinder their adoption [36,37]. Moreover, the growing demand for energy-intensive AI models, such as large language models (LLMs), in data-driven applications underscores the importance of addressing energy consumption as a critical requirement in data management and sharing [38].

A *trust framework* is essential to manage data in data spaces, allowing secure sharing, identity authentication, and policy enforcement [1,2,11,39]. But decentralization makes uniform management of identities, tracking of provenance, and usage control over systems challenging [40]. Furthermore, distributed ledger technologies present issues with system interoperability and scalability across various infrastructures, even though they provide advantages like transparency and traceability [41]. Because of these complications, trust becomes a barrier to open and safe data management and sharing in data spaces.

*Business models* form an economic base for sustainable data sharing since they support value-based exchange processes, commonly called data monetisation practices [3,42]. Effective business models are essential for enabling trusted and sustainable data sharing across stakeholders in data spaces [1,2,11,43]. They deliver economic incentives and governance structures that encourage data providers to join, set terms of access and usage, and guarantee equitable compensation. In the absence of good business models, data sharing is still ad hoc, not scalable, and simply not economically sustainable, especially in ecosystems involving sensitive, valuable, or commercially significant data [44].

### 2.2 EU projects for addressing data management and sharing

To carry out our analysis, we selected a group of recent EU-funded projects running between 2022 and 2025. These were funded under the Horizon Europe call *HORIZON—CL4–2021-DATA-01–03, “Technologies for data management (AI, Data and Robotics Partnership),”* which specifically aimed to advance technologies for data management, including improving usability, discoverability, interoperability, data quality, energy efficiency, and trust. As Horizon Europe supports research and innovation projects of a scientific nature, this distinguishes the analysed projects from those funded under the Digital Europe programme, which focuses on the concrete conceptualisation and deployment of data spaces. The projects we examined are enRichMyData [45], DataBri-X [45], Green.Dat.AI [46], SEDIMARK [47], STELAR [48], and WATERVERSE [49].

Our selection was guided by two main reasons. First, each project includes at least one co-author of this paper who is directly involved in its implementation. This allows us to offer insights based on first-hand experience and active participation in the work carried out. Second, all six projects focus on data space solutions and have been running long enough to deliver tangible results and lessons learned. Rather than aiming to cover the entire spectrum of ongoing activities in this fast-moving and widely discussed field, we focus on a set of relevant and timely examples that help us draw out strategic insights. These projects are not exhaustive, but we consider them sufficiently representative of the current state of data space development. Their analysis has helped us identify both common approaches and key differences, which we explore further in Section 4.

In this sense, we can highlight that all the examined projects: (a) address fundamental data management and sharing challenges and the requirements derived from them according to the 2020 European Strategy for Data; (b) provide publicly accessible documentation (websites, deliverables, publications) outlining their approach; and (c) span a wide range of technical approaches and field application areas overall. Furthermore, these six projects are a combination of domain-focused and cross-domain projects. These consist of two projects with general-purpose data space toolboxes (enRichMyData and DataBri-X), one for energy-efficient data analysis (Green.Dat.AI), one for a decentralised marketplace for data (SEDIMARK), and two domain-specific ones (STELAR for agriculture and WATERVERSE for water management). To identify the technological solutions associated with these requirements, we examined reference architectures, demonstrators, and technical deliverables from each project. This analysis enabled us to understand how the proposed solutions support the implementation of data spaces in practice.

### 2.3 Conceptual framework for data management and sharing in data spaces

**Fig. 1:**
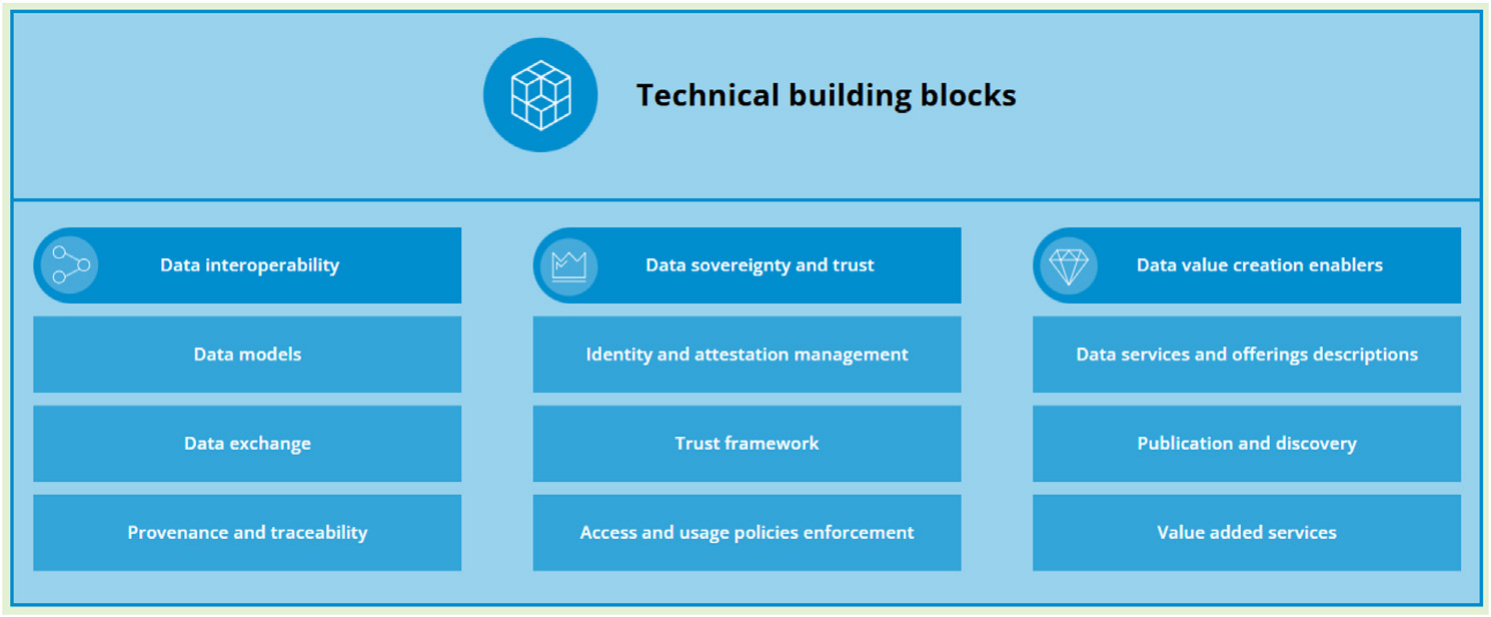
Technical building pillars and blocks for data space realisation - DSSC Blueprint v2.0.

To systematically analyse how current initiatives satisfy the requirements of data management and sharing, we propose a conceptual framework wherein the seven requirements identified in Section 2.1 are harmonized with the three pillars of the DSSC Blueprint for data spaces [11] (Fig. 1). The DSSC provides a minimum viable architecture for deploying data spaces based on three central pillars. These pillars address the practical requirements for functional, governance, and value-creation requirements. The first pillar is *Data Interoperability*, enabling effective exchange and understanding of data between diverse systems using standardised formats, APIs, ontologies, and data provenance capabilities. The second pillar is the *Data Sovereignty and Trust,* which gives data owners control over their assets by applying identity, access, and usage policies. Lastly, *Data Value Creation Enablers* are focused on facilitating the economic and social value of data sharing using services such as catalogues, discovery systems, Artificial Intelligence (AI) workflows, and business models.

**Fig. 2:**
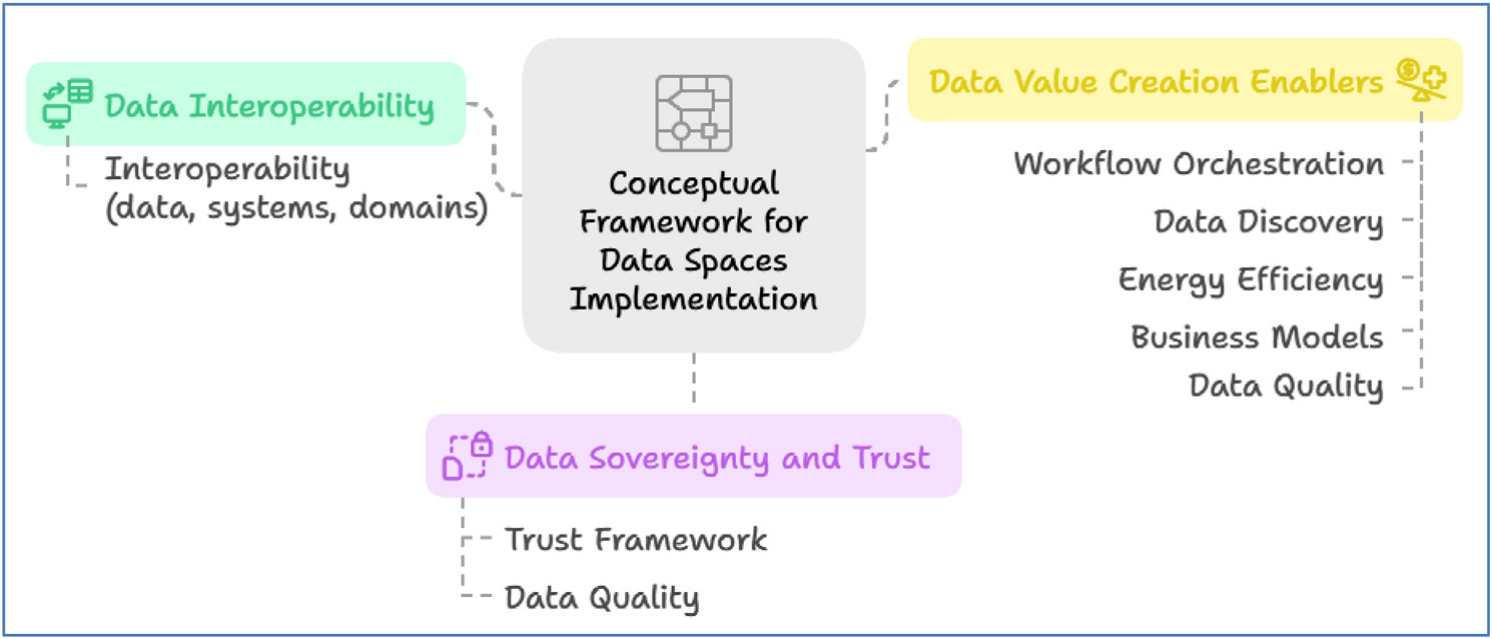
The proposed conceptual framework for data-space implementation shows the alignment between key data management and sharing requirements and the DSSC pillars, including their relevant technical building blocks as defined in the DSSC Blueprint [11].

The proposed conceptual framework (see Fig. 2) establishes a structured connection between the core requirements of data management and sharing, as defined in Section 2.1, and the three foundational pillars of the DSSC Blueprint. Each requirement is assigned to a particular pillar according to the governance and functional mechanisms needed to handle it successfully (see Table 2). For instance, because the *Data Interoperability pillar* relies on standardised communication protocols, APIs, data formats, standardised *data* models (e.g., FIWARE Models [50]) and shared semantic models (e.g., domainspecific models [51]) to facilitate smooth data exchange across diverse systems and domains, the interoperability requirement is intrinsically linked to it.

**Table 2:** Mapping framework table for data spaces implementation.

Requirement	Mapped DSSC Pillar	Explanation
**Interoperability**	Data Interoperability	Requires technical, semantic, organizational, and legal interoperability through APIs, shared vocabularies (e.g., DCAT [52]), data models, and standardised protocols (e.g., NGSI-LD [53]).
**Workflow Orchestration**	Data Value Creation Enablers	Workflows streamline data pipelines and AI services, supporting automation, Continuous Integration and Continuous Development (CI/CD) of stakeholder tools.
**Data Quality**	Data Sovereignty and Trust, and Data Value Creation Enablers	High data quality enhances trust, supports accurate analytics, and boosts the value and reliability of shared data assets.
**Data Discovery**	Data Value Creation Enablers	Metadata management, catalogues, profiling tools, and search interfaces support the discoverability and usability of datasets.
**Energy Efficiency**	Data Value Creation Enablers	Efficient data processing (e.g., federated learning, edge AI) reduces the environmental footprint of data space operations.
**Trust Framework**	Data Sovereignty and Trust	Trust mechanisms (e.g., IAM, blockchain) ensure secure identity verification, policy enforcement, and data sovereignty.
**Business Models**	Data Sovereignty and Trust	Monetisation strategies and collaborative business models (e.g., Data as a Service, tokenization) are enabled by access policies and trust.

Also, the *pillar* on *Data Sovereignty and Trust* is where requirements such as the *Trust Framework* and *Data Quality* are best located. With a perspective towards creating a safe, open, and trusted environment for the sharing of data, this pillar has tools like usage control, policy enforcement, and identity and access management (IAM). Since it supports the dependability of shared information and the standing of data providers, high data quality is also essential to trust.

The *Data Value Creation Enablers pillar* closely relates to requirements concerning *Workflow Orchestration, Data Discovery, Energy Efficiency*, Data Quality and *Business Models.* By ensuring high-quality data, enabling efficient processing, optimizing resources, and supporting sustainable economic models for data exchange and reuse, these components collectively focus on generating concrete value from data. Table 2 provides a detailed mapping between the DSSC elements and the identified data sharing requirements, serving as a blueprint for evaluating approaches and technologies in data space implementations. We adopt this model to present the approach of each project (Section 3) and to enable a structured comparison across all projects (Section 4).

## 3 Overview of six EU projects implementing tools for data spaces

In this section, we analyse how the selected projects satisfy the requirements defined in the proposed data management and sharing conceptual framework, and we highlight the core technologies and methodologies they adopt. We also present a representative use case for each project, highlighting the domain needs it addresses.

### 3.1 enRichMyData: enabling data enrichment pipelines for AI-driven business products and services

**Fig. 3:**
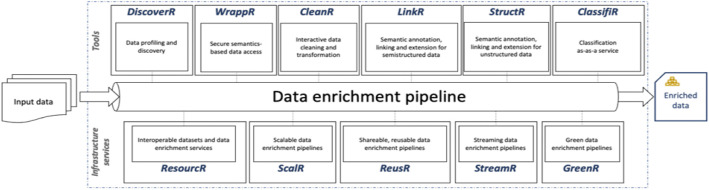
The enRichMyData’s reference architecture.

The enRichMyData project [45] is building an open-source toolbox [54] to simplify data enrichment for AI and Big Data. It addresses the time-consuming process of finding, cleaning, and combining data, commonly referred to as the data enrichment pipeline, before analysis can begin. Its modular tools produce FAIR-compliant datasets and support their reuse within data spaces. Each tool in the enRichMyData toolbox (Fig. 3) is purpose-built to tackle one of the core requirements in data sharing ecosystems. To support **Interoperability**, the tool *WrappR* creates a virtual semantic layer on top of existing data sources, exposing them as *Knowledge Graphs*. This functionality allows for federated, secure access via *SPARQL* queries and semantic inference. In support of **Data Discovery**, *LinkR* connects datasets to ontologies, enhancing semantic annotations and *ML techniques*, while *ReuseR* provides search and recommendation functionality. *DiscoverR* also supports querying data catalogues through *semantic profiling*, which aids in the simplicity of the user's ability to determine the correct datasets for their needs. Semantic alignment across domains is crucial, and these capabilities directly support the findability and reusability principles mandated by data spaces [33,35]. *CleanR* offers an interface for data transformation and cleaning to enhance **Data Quality**. It offers AI-assisted suggestions for data preparation, standardization, and correction to ensure that shared data meets quality thresholds necessary for reuse in collaborative ecosystems. Moreover, document-level taxonomic, custom label, and industry-standard-based data classification is supported by the *ClassifiR* tool to further facilitate data quality assessment. **Workflow Orchestration** is also addressed by *ScalR* and *StreamR*, which support scalable data processing in cloud and on-premises environments, including real-time and batch workflows. These tools allow users to design enrichment pipelines that operate within the distributed environments typical of data spaces.

To enable a **Trust Framework**, enRichMyData uses *WrappR* and *ReuseR* to manage data access with public/private settings, sharing, versioning, and integrity controls for secure collaboration. In addition, the toolbox includes *GreenR*, an **Energy Efficiency** monitoring instrument, that helps with monitoring and optimising energy consumption in enrichment procedures, allowing in this way the sustainability goals to be more aligned with AI needs for data spaces. A key feature of enRichMyData is that it is reusability-focused, enabling the sharing of enriched datasets as well as transformation logic to enable collaboration and allow maximum exploitation of shared data.

A representative use case for enRichMyData is found in the automotive manufacturing domain. In the context of the Catena-X alliance [55], the project has developed an automated pipeline to prepare heterogeneous robotic welding data for AI analytics. This addresses common manufacturing data challenges: vast volumes of heterogeneous sensors’ and processes’ data must be cleaned, integrated, and semantically aligned before analysis. Previous studies show that manufacturing supply chains often suffer from incompatible data formats and low interoperability, and thus, limited data sharing capabilities hinder end-to-end visibility and adaptive control [56]. By automating data enrichment with enRichMyData (using tools like *CleanR, LinkR,* and *StructR*), enRichMyData reduces manual effort and unlocks underutilized data. This is achieved by transforming raw tables and documents into structured *Knowledge Graphs*. Better data quality and interoperability in the automotive domain, therefore, allow for more efficient AI-driven quality control in welding, which leads to production increase and efficiency. Thus, advanced data enrichment pipelines in the data spaces’ environment pave the way for more extensive data monetisation and intelligent manufacturing techniques in the automotive sector [57].

### 3.2 DataBri-X: building modular tools for interoperable and trustworthy european data spaces

**Fig. 4:**
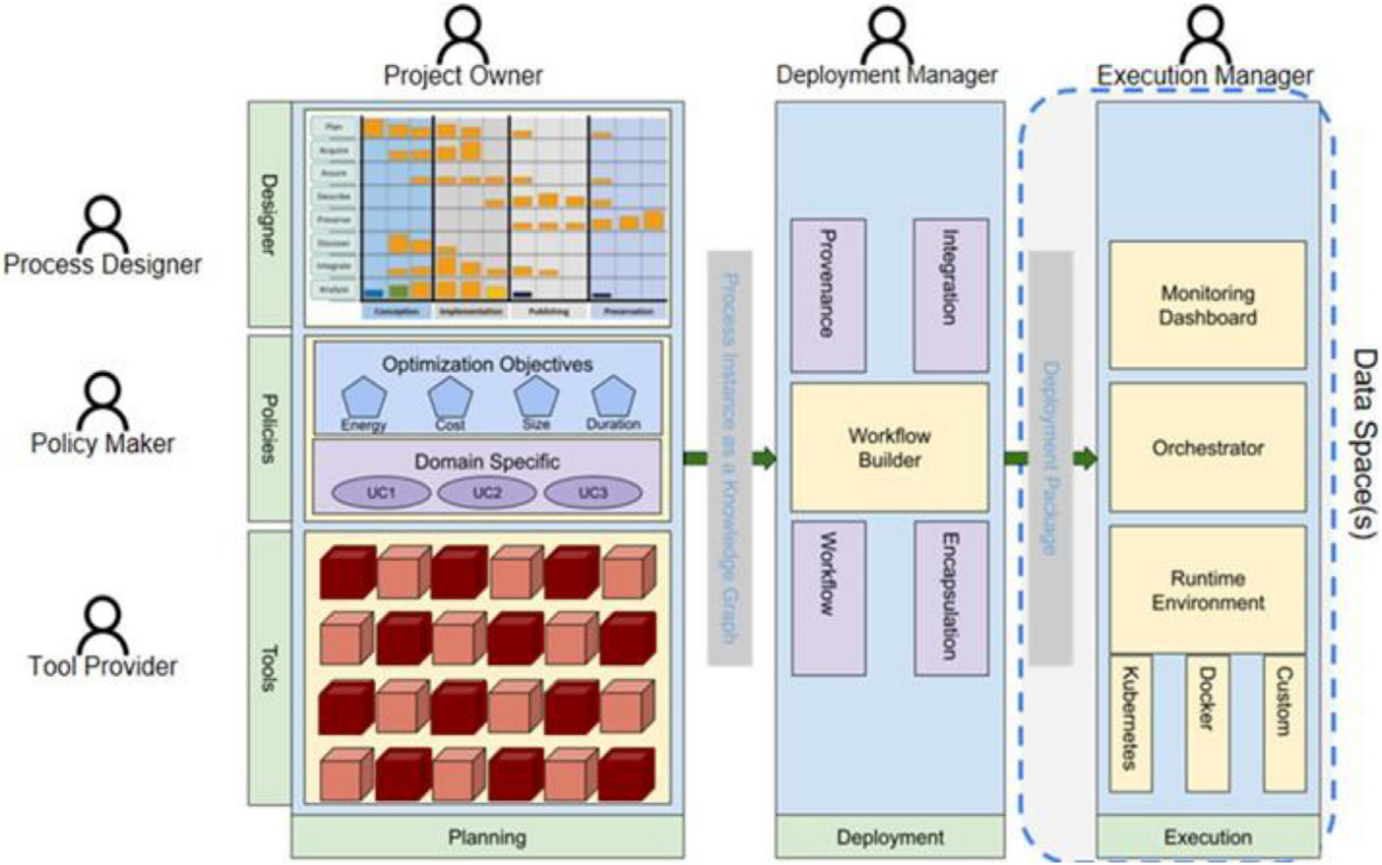
The DataBri-X’s reference architecture.

DataBri-X [58,59] offers a modular and easily navigable toolbox of “bricks” to help create and integrate data spaces (Fig. 4). The complex integration of multiple components, including storage, metadata handling, interoperability, and security, is often necessary when building a data space. DataBri-X offers a ready-to-use solution to expedite this process [60]. In particular, DataBri-X enhances **Interoperability** by utilizing *Semantic Web standards***.** It uses the *DCAT vocabulary* [52] to describe datasets, and the *SOSA ontology to* describe time-series and sensor data [61]. Additionally, a common vocabulary is created for all lifecycle tools to guarantee semantic consistency throughout the data pipeline. *IDS Connectors* also ensure compatibility across the involved stakeholders [62]. DataBri-X offers a process-oriented approach to **Workflow Orchestration** through *JenPlane* [63], a dynamic governance layer that automates the setup and optimization of data workflows. This orchestration engine selects and combines tools to enable adaptive execution that can be modified to satisfy energy, performance, or policy constraints based on semantic annotations. To enhance **Data Quality**, the toolbox includes validation and profiling modules that analyse and refine datasets throughout the ingestion and publication phases. These tools help ensure that shared data assets across data spaces meet accuracy and integrity requirements. **Data Discovery** is enhanced in DataBri-X through the offering of recommendation engines and the capability of semantic search. Both characteristics help to gain further data assets (i.e., datasets) and corresponding insights (i.e., analytics). **Energy Efficiency** is one of DataBri-X's primary goals, and it is achieved through dynamic workflow optimization. The *JenPlane* engine reconfigures processes to use less energy without compromising performance. Regarding the **Trust Framework** requirement, DataBri-X fully conforms to the *IDS Reference Architecture Model (RAM)* [62]. Thus, it utilises IDS-compliant service gateways (also known as *IDS Connectors*) as a fundamental step in enabling communication and trust between data providers and consumers. These *Connectors* ensure that data is exchanged only under agreed-upon policies, with secure authentication mechanisms. Finally, data usage control is applied, specifying who can access what, under which conditions, and for what purpose via policies. Accordingly, DataBri-X in its orchestrator layer (Fig. 4) enforces these policies to ensure data sovereignty is preserved even after sharing.

A key use case for DataBri-X lies in the energy domain, specifically creating “renewable energy communities” (RECs). The EU’s “Clean Energy for All Europeans” directive promotes citizen-driven RECs, but creating them faces data challenges: diverse stakeholders (homes, generators, grids) must share energy profiles, device data, and asset information, yet no standard formats or tools exist for visualizing and managing this data [64]. The role of REC is to provide tangible benefits to citizens, such as improved energy efficiency and reduced electricity costs. Interoperable data formats and sharing guidelines are “highly needed for interoperability and the widespread deployment” of energy communities [65], and DataBri-X directly targets this gap through the adoption of the data spaces paradigm. DataBri-X delivers simulation tools under the REC use case to assess various community configurations across disparate data sharing situations, assisting stakeholders in analysing trade-offs and viability. By enabling the preparation and semantic harmonization of asset information in readiness for community integration, it also expedites the onboarding process for participants. Communities can be both well-designed and operational through a two-stage process. DataBri-X contributes to the EU's decarbonization objectives by facilitating the secure exchange, searchability, and standardization of energy-related data within data spaces [12]. This facilitates energy trade and collective energy management.

### 3.3 Green.Dat.AI: energy-efficient AI-ready data spaces

**Fig. 5:**
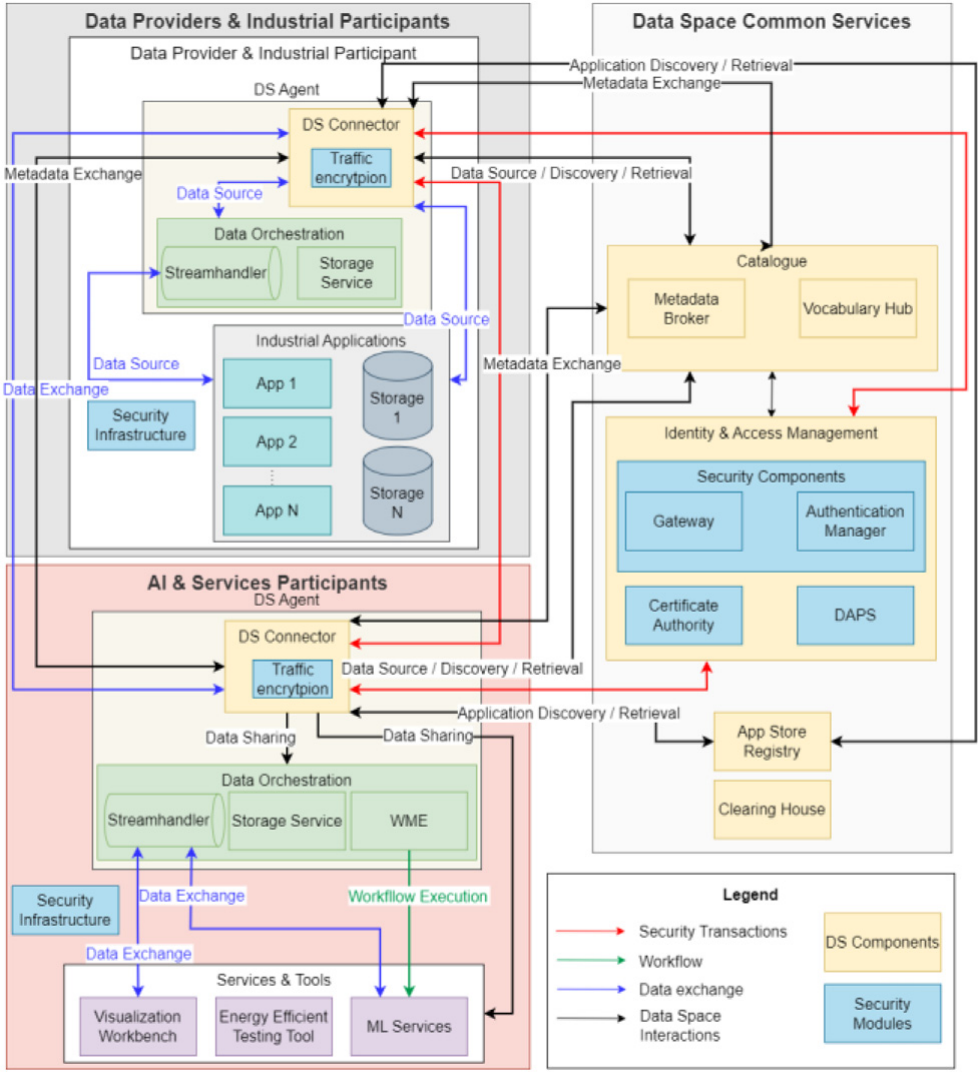
The Green.Dat.AI-’s reference architecture.

The Green.Dat.AI project [46,66] leverages AI and data spaces to support the green transition, introducing a data-space-ready reference architecture (Fig. 5) and a toolbox of reusable, energy-efficient AI services, tested in a large-scale data management framework [34]. Green.Dat.AI addresses the requirement of **Interoperability** by integrating semantic technologies via a *Vocabulary Hub*. This component manages common vocabularies to ensure effective communication across diverse systems and data formats by utilising *EDC IDS Connectors* (*Sovity Edition* [67]). Green.Dat.AI’s *Workflow Management Engine* [68] automates the design, management, storage, and execution of AI workflows for data space participants, using open-source tools like *Apache Airflow* [69] for scalable *Directed Acyclic Graphs (DAG)* **Workflow Orchestration**. The project enhances **Data Quality** by implementing synthetic data generators, assessed using *Root Mean Square Error (RMSE)* and validated by domain experts for alignment with real-world patterns, particularly through spatial validation of location and traffic distributions. In addition, **Data Discovery** is enabled through the *Catalogue Component* (Fig. 5), as part of the Data Space Common Services. The *Metadata Broker* enhances discovery by managing metadata through *Self-Descriptions*, summarizing participants and *Connectors* for seamless data exchange. **Energy Efficiency** is addressed in Green.Dat.AI through the *Energy Efficiency Framework***,** a component that monitors the energy consumption of *ML models*, providing standardised and measurable evaluation of corresponding AI algorithms, assessing key metrics such as energy consumption, model accuracy, data volume, and execution time [62]. The project provides a **Trust Framework** with a *Certificate Authority (*CA*)* providing digital identities and a *Dynamic Attribute Provisioning Service (DAPS)* to strengthen participant identities and *Connectors*. It supports Identity and Access Management with a *Gateway* for API traffic, load balancing, rate limiting, and an *Authentication Manager.* Other *security components* include an Internet of Things (IoT)-enabled *Network Intrusion Detection System (NIDS)* and a *Trusted Execution Environment (TEE)* to ensure secure data exchange, remote attestation, and encryption of memory to establish trust between data providers and AI services. Finally, Green.Dat.AI implements **Business Models** rewarding data sharing with a *monetary model* that compensates data providers for the supply of data for regression jobs and a *non-monetary model* to exchange similar datasets. These transactions are facilitated by a *blockchain-based system* (e.g., *Predico*) for secure, traceable transactions [70].

Green.Dat.AI's “Smart Farming” use case for agriculture combines data and AI to make crop management more efficient. It demonstrates how drone surveys, in-situ sensors, and satellite imagery can be combined in a data space environment to provide real-time maps of fertilization, early pest detection, and predictions of soil health and harvest times. This approach reduces chemical inputs and improves yields. In addition, by embedding *digital twins* of field advisors and existing industrial applications, Green.Dat.AI can simulate different data management strategies with minimal training data [71]. This pilot leverages data spaces’ components (i.e., data-catalog (*Piveau* [72] and *EDC Connectors* [67]) for interoperability, and strong security components (i.e., based on *Keycloak* [73], *OAuth2* [74]) for enabling trust and data sovereignty [75]. This use case tackles known agriculture data challenges: farm data is highly heterogeneous and often siloed, and many farmers hesitate to share data due to privacy concerns [75]. By demonstrating integrated, energy-efficient analytics coming from AI services and clear data governance (via the data orchestration components), Green.Dat.AI exemplifies how smart farming can overcome these barriers and create value from shared data in a data space environment.

### 3.4 SEDIMARK: secure decentralised intelligent data MARKetplace

**Fig. 6:**
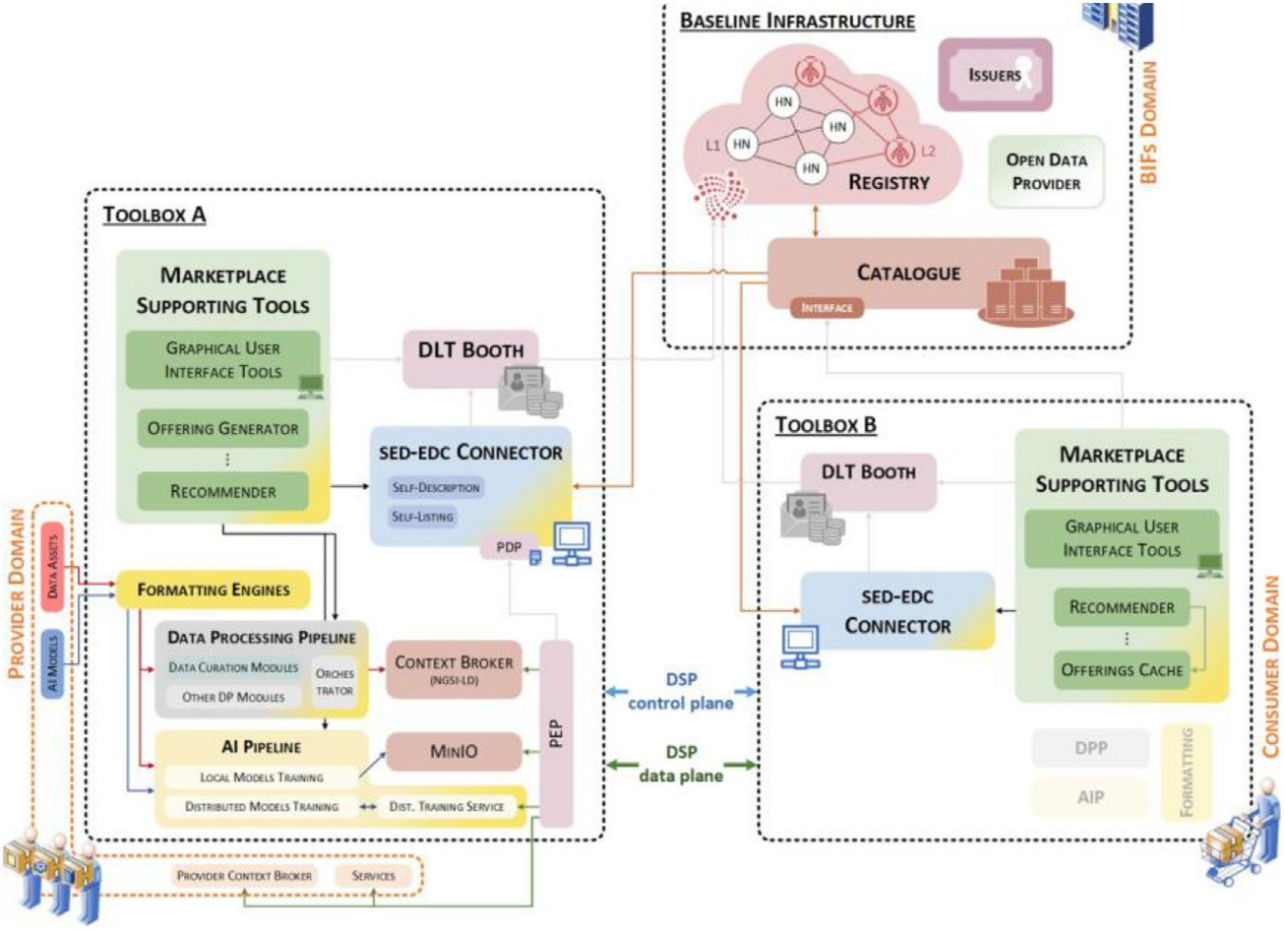
SEDIMARK’s reference architecture.

SEDIMARK [47,76] aims at building a data and services marketplace based in the context of data spaces. The marketplace encompasses the toolboxes [77] with which data providers and consumers are able to use a baseline infrastructure providing the substrate for marketplace processes (Fig. 6). To ensure **Interoperability** SEDIMARK defines a common information model (*Marketplace Information Ontology***)** that extends the *DCAT AP* [52] *ontology* for asset metadata by incorporating properties from established schemas like the W3C *Data Quality Vocabulary (DQV)* [78] and W3C *Open Digital Rights Language (ODRL)* [79]. Moreover, it leverages *NGSI-LD* [53] to promote interoperability also at the data level. Furthermore, technical interoperability among marketplace participants is ensured via *IDS EDC Connectors* [67]. In terms of **Workflow Orchestration**, SEDIMARK provides an *AI pipeline***,** a system composed of interconnected components designed for training or optimizing *ML models* and providing inference and analytics supporting both local and distributed training via the corresponding modules: the *Local Model Training (LMT)* and *Distributed Model Training (DMT)* module [80]. To assess and improve **Data Quality,** SEDIMARK introduces the *Data Processing Pipeline (DPP),* a collection of tools for evaluating and curating data. These tools keep common information model compliance and perform functions such as profiling, deduplication, outlier discovery, missing-value imputation, and enrichment [81]. To facilitate **Data Discovery,** *Catalogues* are built as indexed, searchable views of a *Registry***,** which is also provided to facilitate and promote offerings’ discoverability in the marketplace. The baseline infrastructure supports multiple catalogues, enabling scalable and federated discovery across domains. **Energy Efficiency** is tackled in the *AI enabler component* by incorporating techniques that focus on reducing the energy consumption associated with training and deploying *AI models*. The **Trust Framework** in SEDIMARK is enabled through a *MarketPlace Wallet*, which ensures the secure storage of essential credentials, including authentication tokens and keys, enabling users to interact among themselves within the marketplace securely and seamlessly. The used digital identity model follows the *W3C Self-Sovereign Identity (SSI) model* [82], which uses *Distributed Identities (DIDs)* to give users and devices full control of their identity data. Trust is further increased by the *Registry***,** which is achieved via *Smart Contracts* to be able to handle the entire life cycle of the asset (data/service) throughout the marketplace. Finally, SEDIMARK introduces flexible **Business Models** that include both *monetized transactions* and *non-monetary sharing options*, creating a decentralised data marketplace where data providers can be rewarded for sharing their assets. The business models are based on *Smart Contracts* and the combination of *Fungible* and *Non-Fungible Tokens (NFTs).*

One of SEDIMARK’s targeted use cases is in urban mobility data. Two pilot scenarios include bicycle usage planning in Santander, Spain, and a mobility digital twin in Helsinki, Finland, illustrating its benefits. In Santander, the city has expanded bike lanes but needs detailed usage patterns and origin-destination data to plan new routes and parking. SEDIMARK allows diverse stakeholders (municipal sensors, citizen apps, private fleets) to contribute and semantically enrich data. Its *AI pipelines* can validate, link, and transform bike-sharing and traffic data under fine-grained privacy policies (public vs. commercial data), enabling authorized planners to analyse “black spots,” predict demand, and optimize infrastructure investments. In Helsinki, an existing 3D city model can be linked with mobility flows (goods and people’s movement). Normally, such multi-source data is siloed, and concerns about location privacy and competitive sensitivity are major barriers to sharing [83]. SEDIMARK overcomes this by enforcing privacy-by-design policies and cryptographically securing credentials so that only vetted users can access sensitive maps. In both cases, the marketplace model shifts traditional norms: city councils can remain data providers while private actors (e.g., mobility startups) contribute their data in exchange for insights. The trust mechanisms and visual analytics, along with the established data space in SEDIMARK, facilitate collaboration that was previously inhibited by privacy, security, and governance issues [83].

### 3.5 STELAR: spatio-temporal linked data tools for the agri-food data space

**Fig. 7:**
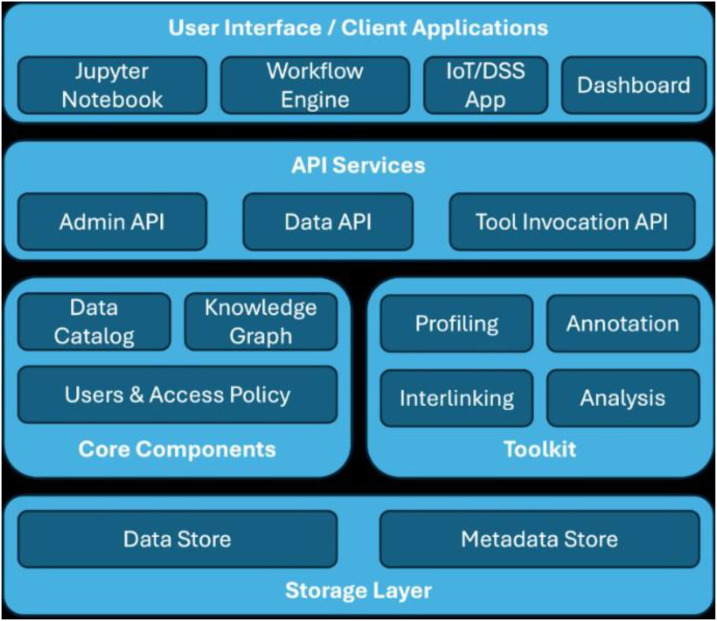
The STELAR’s reference architecture.

STELAR project [48,84] aims to design and implement an innovative Knowledge Lake Management System (KLMS) to support the European agri-food data space [85]. To address **Interoperability**, STELAR leverages *Linked Data* and *Semantic Technologies*, offering *entity linking* algorithms as well as *schema* and *ontology matching* techniques to ensure seamless data integration and reuse. Interoperability between data space participants can be achieved using *IDS Connectors* [62,67]. In STELAR, datasets are processed through workflows that can be supported by the proposed reference architecture (Fig. 7) via a **Workflow Orchestration** engine capable of capturing metadata about multi-step workflows. **Data Quality** is supported in STELAR via the *Data Profiler* component, which is used to identify and mitigate relevant issues, including anomaly detection and missing values imputation. To enable **Data Discovery**, STELAR provides a *Data Catalogue* and a corresponding *API* for publishing and searching datasets. Additionally, a *Knowledge Graph* is used to enhance metadata search and support entity interlinking, further facilitating data discovery. Finally, to enhance **Energy Efficiency**, STELAR introduces an engine for constructing and using a wide range of *data synopses.* These synopses act as compact summaries that reduce the need for extensive data processing and transfer when handling large data volumes. The synopses are integrated into a *processing engine* that supports parallel and scalable computation, which is further optimized and customised to accommodate various data types and use cases.

One specific use case of STELAR is forecasting early-season crop development from the agricultural community. Farmers and policymakers need early yield estimates to plan inputs and food security, yet existing forecasts issue them too late. Thus, STELAR combines heterogeneous Earth Observation data (multispectral and Synthetic Aperture Radar (SAR) satellites, weather forecasts, etc.) with *deep learning* techniques to identify crop type and condition even before vegetation is visibly detectable. This entails handling very large, multi-source data (radar and optical, varying resolutions, spectral bands), and gap filling (e.g., cloud blocking imagery) through sensor fusion. STELAR tools allow for data stream selection, harmonization, and fusion: e.g., missing optical data is filled with radar or alternative satellites. This tackles smart farming system technical issues: bringing together and processing decentralised, heterogeneous datasets within reasonable time windows. The project illustrates that a data space can solve common agri-data challenges such as system heterogeneity and farmers' mistrust, issues that have been proven to be major barriers to data sharing in agriculture. STELAR delivers with its approach clear data ownership, standards for interoperability, and control over quality while enabling actionable insights for crop management [75].

### 3.6 Waterverse: water data management ecosystem for water data spaces

**Fig. 8:**
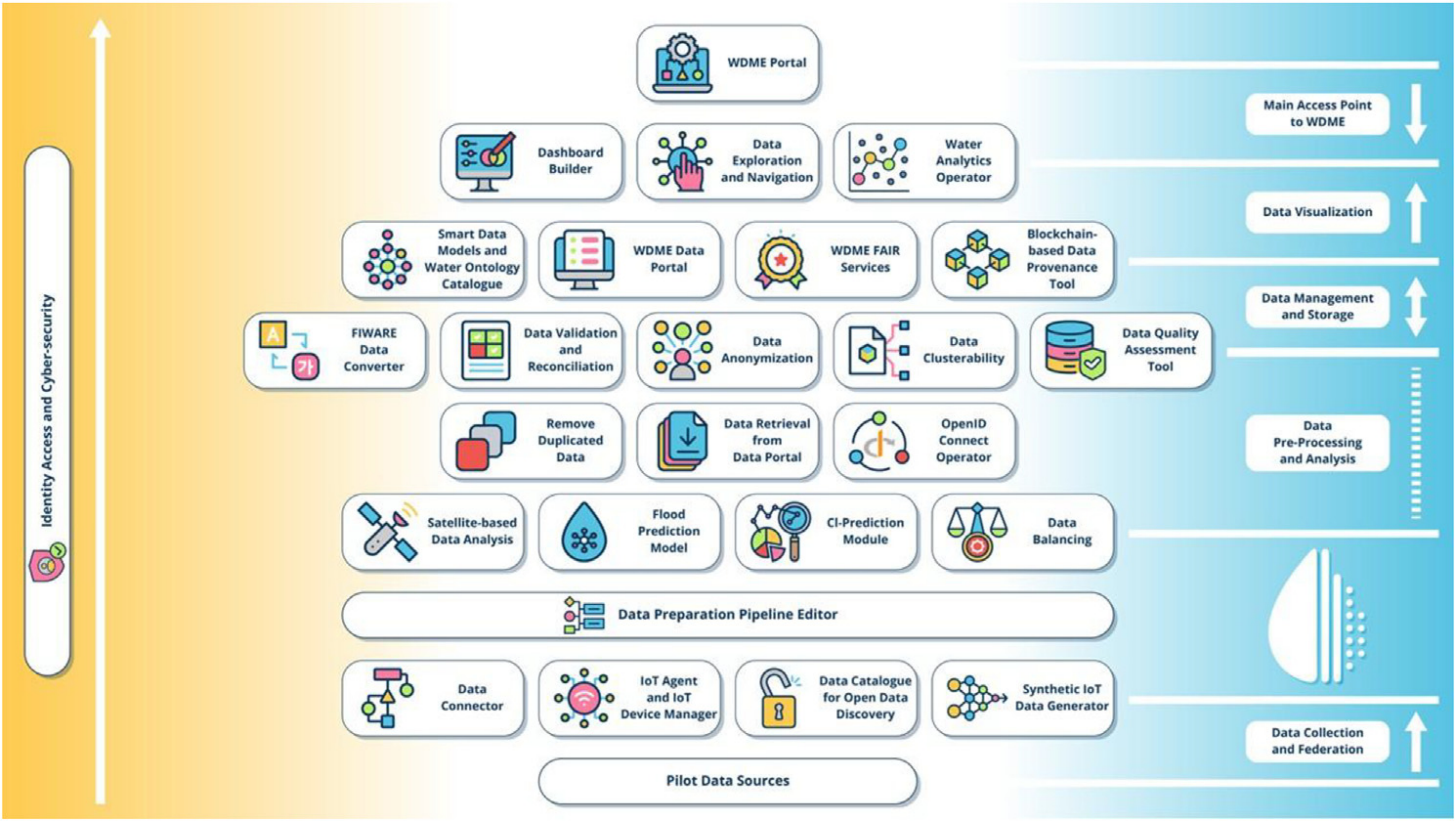
The WATERVERSE’s reference architecture.

The WATERVERSE project [49,86] focuses on the water domain and aims to create a Water Data Management Ecosystem (WDME) to facilitate the digital transformation of water utilities by applying data spaces technology and a multi-layer architecture (Fig. 8). The established water data space supports the resilience of water utilities through several data-sharing scenarios. **Interoperability** is addressed in WATERVERSE through the *DCAT-AP catalog* [52], which incorporates FAIR principles and the *MELODA 5* dimensions [87] and *FIWARE Data Space Connectors* [88]. Also, the WDME provides data ingestion from IoT and remote sensing sources, with a harmonization layer (e.g., mapping to *FIWARE Smart Data Models* [50] /*NGSI‑LD* [53]) for semantic interoperability. **Workflow Orchestration** is supported in WATERVERSE through the *Data Preparation Pipeline Editor (DPPE)*, which allows users, including non-experts, to easily design data streams, called **"***Mashups***"**, through a graphical interface. Mashups consist of inputs, JSON-formatted outputs [89], and logical operators that perform tasks such as harmonization, validation, and processing. To enhance **Data Quality,** the *Data Quality Assessment (DQA) Tool* has been developed and integrated into the WDME, providing insights across five key metric dimensions: completeness, consistency, identifiability, timeliness, and validity. Moreover, additional tools, namely *the Data Validation and Reconciliation (DVR) Tool* and the *Data Balancing Tool (DBT)*, work together to assess and improve dataset quality by detecting anomalies, duplicates, missing values, and imbalanced classes. WATERVERSE enhances **Data Discovery** by integrating multiple open data portals through a centralised platform that leverages the *CKAN platform* [90] for managing and federating datasets. It also incorporates the *IDRA platform* [91] to import and manage metadata from various Open Data management systems, providing standardised access, multilingual search capabilities, and support for *SPARQL* queries, *DCAT-AP standards* [52], and third-party applications for data visualization. Energy efficiency optimisation is addressed by monitoring and enhancing energy-intensive water data management processes within the *DPPE pipelines,* leveraging the open-source *Prometheus monitoring tool* [92,93]. A **Trust Framework** is established in WATERVERSE through Identity Access Management and the adoption of relevant technologies (*OpenID protocol* [94], *OAuth2* [74], *Keycloak* [73]). Additionally, the *Blockchain-based Data Provenance tool* which relies on the *Distributed Ledger Technology (DLT)* using *AEI Smart Contract* (*ERC721 standard* [95]) and the *CanisMajor blockchain adaptor* [96], allows the provenance and traceability of data. WATERVERSE focuses on leveraging water-related data to create economic value while enhancing data management practices in the water sector. Thus, *data monetisation schemes* (e.g., monetizing data analytics) constitute the basis for establishing **Business Models** for WATERVERSE’s participants.

WATERVERSE’s main use case aims at predicting water quality changes throughout the processes of the established WDME that support different *DPPE pipelines*. Rapid urbanization stresses water supplies and raises the risk of pollution, and in fact, operators need to forecast quality issues (e.g., contamination events) prior to treatment to maintain public health and reduce costs. WATERVERSE blends sensor data (turbidity, pH, flow) and historical logs to provide predictive models of water quality for every stage of treatment. This deals with common issues: water utility data is isolated in *SCADA (Supervisory Control and Data Acquisition) systems* and *GIS maps*, and interoperability issues prevent generating meaningful analytics. Indeed, smart water management suffers from “data interoperability issues” and needs cross-sector data-sharing standards [97], and WATERVERSE addresses this gap. By unifying water-related data in a common platform with standardised schemas, WATERVERSE enables more reliable predictions and process optimisation. The platform’s federated catalogue ensures that diverse data (from sensors, weather, and infrastructure databases) can be discovered and queried consistently, while the enabled trust framework allows utilities to share sensitive data securely. In doing so, WATERVERSE addresses the critical interoperability and governance gaps that have limited prior water data initiatives [97], ultimately enhancing decision-making and sustainability in the water domain by implementing a domain-specific data space (in water).

## 4 Cross-project analysis

Based on the information about the different projects, we have then conducted a cross-project analysis using the proposed framework, identifying commonalities and differences (subSection 4.1), and open research topics that warrant further investigation (subSection 4.2).

### 4.1 Approaches and technologies

At first glance, the comparison shows that, with the notable exception of business models (see Table 3), all of the projects under consideration satisfy the most of the identified data management and sharing requirements. Since these requirements are defined as necessary conditions for overcoming the underlying challenges reported in European policy and research, this emphasizes a twofold necessity. On the one hand, the interrelation between the derived requirements leads to the need for addressing them in a holistic manner. On the other hand, a need to investigate collaborative business models that go beyond conventional data monetisation techniques. Our results for each requirement are summarized below, and Table 4 gives an overview of the technologies used in the projects.

**Table 3:** Summary of Projects’ offerings for identified requirements for Data Management and Sharing.

Requirements	enRichMyData	DataBri-X	Green.Dat.AI	SEDIMARK	STELAR	WATERVERSE
*Interoperability*	*	*	*	*	*	*
*Workflow Orchestration*	*	*	*	*	*	*
*Data Quality*	*	*	*	*	*	*
*Data Discovery*	*	*	*	*	*	*
*Energy-Efficiency*	*	*	*	*	*	*
*Trust Framework*	*	*	*	*		*
*Business Models*			*	*		*

**Table 4:** Technologies used for identified requirements for data management and sharing.

Project	Interoperability	Workflow Orchestration	Data Quality	Data Discovery	Energy Efficiency	Trust Framework	Business Models
**enRichMyData**	WrappR, Knowledge Graphs	ScalR & StreamR for distributed enrichment pipelines	CleanR and ClassifiR tools for AI-assisted data preparation	DiscoverR, LinkR, and ReuseR for semantic search recs	GreenR monitors enrichmt ‘s energy	Access controls via WrappR, ReuseR	N/A
**DataBri-X**	DCAT, SOSA, and shared vocabularies, IDS EDC Connectors	JenPlane auto-configu res workflows by constraints	Profiling and validation modules on ingestion /publishing	Semantic search and recommendatio n tools	JenPlane optimises workflows for energy efficiency	IDS approach based on usage control	N/A
**Green.Dat.AI**	Vocabulary Hub, IDS EDC Connectors	Apache Airflow-base d Workflow Mgmt Engine	Synthetic data generation and expert validation	Catalogue, Metadata Broker,self-desc riptions	Energy Framewk., evaluating AI model efficiency	CA, DAPS, TEE, NIDS (security compone nts)	(Non) Monetary Models, Blockchai n
**SEDIMARK**	NGSI-LD & DCAT-AP ontologies, DQV and ODRL, IDS EDC Connectors	AI pipeline modules (LMT/DMT) for training	Data Processing Pipeline (DPP) with deduplication, profiling, etc.	Federated graph catalogues & DLT registry with indexed views	Energy-sav ing strategies in AI training modules	SSI, DIDs, smart contracts, NFT-based	(Non) Monetary Transactio ns, NFTs
**STELAR**	Schema matching and entity linking, IDS Connectors	Workflow metadata capture (no full orchestratio n)	Anomaly detection and imputation via Data Profiler	Agri-food data catalogue with knowledge graph enhancement	Data synopsis engine for processing /transfer costs	IAM via Keycloak	N/A
**WATERVERSE**	DCAT-AP,FIWA RE/NGSI-LD, Meloda5, FIWARE Connectors	DPPE GUI for designing mashups by non-experts	DQA tool + DVR and DBT assess multiple quality metrics	Federated CKAN portals and IDRA for discovery and reuse	Monitoring metrics for energy efficiency via WDME pipelines	IAM via Keycloak, OpenID Connect, and OAuth2	Data Monetisat ion Schemes

**Interoperability:** All six projects prioritize semantic interoperability using shared vocabularies and data models. While all adopt semantic standards, their implementations vary by domain. DataBri-X and SEDIMARK formalize metadata via *DCAT* and *NGSI-LD*, whereas enRichMyData and STELAR emphasize graph-based knowledge representation. WATERVERSE extends catalogue standards with water-specific FAIR metadata, and Green.Dat.AI focuses on a centralised *vocabulary service* for AI data, part of its offered Data Space Common Services. The common thread is a reliance on *open standards* (*DCAT, NGSI-LD*, etc.) and semantic mappings to enable interlinking and break down data silos. We notice key differences in specific ontologies or schemas each project uses (often domain-driven) and how extensively they build custom models (SEDIMARK’s marketplace ontology vs. others reusing existing vocabularies). Also, mainly *IDS-compliant Connectors* are used to facilitate communication among different data space components: *IDS EDC connectors* are used in DataBri-X, Green.Dat.AI, and SEDIMARK, and are also foreseen in STELAR, while *FIWARE DS Connectors* are used in WATERVERSE.

**Workflow Orchestration:** To facilitate distributed data pipelines, each project offers tools that are mainly *workflow engines*. Although the examined projects facilitate automated pipelines, their scopes and levels of complexity vary. In particular, Green.Dat.AI and DataBri-X provide full-featured workflow engines (based on *Airflow* and *JenPlane*, respectively) that facilitate AI workflows. WATERVERSE prioritizes user-friendly mashup design, while SEDIMARK concentrates on AI model pipelines. STELAR's support is restricted to workflow tracking (metadata), reflecting its emphasis on metadata management rather than full workflow execution or orchestration. We conclude by observing the distinctions outlined above, namely in the orchestration's dynamic or user-friendly nature (code-based vs. GUI-based, static vs. adaptive workflows) and in their focus (AI-centric workflows vs. general ETL pipelines). These distinctions affect who can use the workflows, how adaptable they are, and what kinds of tasks they support.

**Data Quality:** All projects provide data quality-improvement components (e.g., data validation, data accuracy), but with different emphasis. enRichMyData and DataBri-X emphasize generic cleaning and profiling. Green.Dat.AI’s approach is to enable synthetic data generation with domain validation. WATERVERSE focuses on multi-metric assessment of water data. SEDIMARK’s *DPP* is a solid pipeline for marketplace data, and STELAR provides profiling and imputation functionalities mainly for tabular data and time series. Finally, differences can be noted in techniques: e.g., Green.Dat.AI uses synthetic data generation while others focus on cleaning real data. Also, WATERVERSE applies formal metric scoring while enRichMyData provides an interactive cleaning interface. These reflect methodological differences in terms of implementation since some projects embed quality improvement into user workflows (enRichMyData’s UI, WATERVERSE’s dashboards), while others treat it as a behind-the-scenes pipeline process (DataBri-X, SEDIMARK).

**Data Discovery:** Every project facilitates the discovery of data through catalogues, search, or semantic linking by utilizing metadata that can be searched. Semantic linking and recommendations are the main goals of enRichMyData and DataBri-X. SEDIMARK and Green.Dat.AI are dependent on centralised brokers and registries. STELAR uses graph-enhanced search for agricultural data, while WATERVERSE federates external portals in a unique way (using *CKAN*/*IDRA*). In summary, common approaches include the use of standard metadata formats (*DCAT*/*DCAT-AP* in many cases) and providing corresponding user-friendly search interfaces or APIs. The differences lie in how advanced the discovery features are: some projects use *AI/ML* for recommendations (enRichMyData, DataBri-X), others focus on federation of catalogues (WATERVERSE, SEDIMARK). Additionally, domain-specific ontologies (STELAR’s agri-food KG, SEDIMARK’s marketplace schema) tailor the discovery to their context. **Energy Efficiency:** Energy efficiency is addressed in the examined projects through monitoring or optimisation. Thus, all projects consider energy, but in various ways. DataBri-X and enRichMyData focus on workflow reconfiguration, while Green.Dat.AI provides a measurement tool (*Energy Efficiency Framework*). STELAR’s synopsis engine is an innovative data reduction method. SEDIMARK optimises AI model workflows. WATERVERSE acknowledges energy via its architecture so as to assess and visualise metrics such as computational time, data storage, network usage, etc. of energy-intensive data management processes through the mashup editor (*DPPE*). Therefore, we notice a difference in depth of implementation: Green.Dat.AI and DataBri-X explicitly optimise or measure energy in real-time, whereas others provide the means to be efficient (e.g., distribute processing, use synopses) but do not yet have standardised energy metrics.

**Trust Framework:** Most projects implement advanced trust mechanisms. In common, projects handle identity and policies, but differ in sophistication. DataBri-X follows the IDS specifications model for enabling trust [62]. Green.Dat.AI and SEDIMARK go further with dedicated components and relevant technology (e.g., *DAPS, blockchain, SSI* [82]) for enabling more transparency. enRichMyData enforces access within its tools (*WrapR, ReuseR*). WATERVERSE and STELAR use conventional *Identity Access Management tools*. Consequently, we notice several technologies that are used for enabling trust, such as *IDS Connectors* and *blockchain Smart Contracts*, each of them having advantages and disadvantages in terms of scalability and complexity.

**Business Models:** Some projects have explored the development of business models through both monetary and non-monetary schemes. This is the case for Green.Dat.AI, SEDIMARK, and WATERVERSE. A slight difference can be observed in the underlying technologies because this affects the type of incentives. Thus, Green.Dat.AI and SEDIMARK utilize *blockchain-based solutions* such as *Predico* and *NFTs* [70,82], respectively, while WATERVERSE focuses on data monetisation strategies derived from project outcomes, such as data analytics services. In contrast, the other examined projects have not yet begun developing business models. This may suggest that domain-specific business model development in data spaces frequently involves extensive coordination and time investment by numerous diverse actors. Finally, the variety of strategies means that the development of models for a sustainable data economy is a continuing process in the context of developing data spaces.

Across these six projects, we observe a convergence toward a common toolbox of technologies and approaches such as semantic interoperability, workflow automation, data catalogues, identity management, and in some cases, distributed ledger or blockchain that collectively realize core building blocks of data spaces as highlighted in the DSSC Blueprint [11]. We argue that the projects’ complementarity can be seen as an opportunity for further collaboration across data spaces. Thus, a unified approach for implementing data spaces can further accelerate the development of a standardised, interoperable data-sharing environment. By leveraging the strengths and capabilities developed within different projects, we are better positioned to satisfy major data management and sharing requirements. However, a number of underlying challenges that these requirements aim to address are still not fully resolved, indicating crucial areas for additional investigation in upcoming data space projects. We analyze these issues in subSection 4.2 below.

### 4.2 Research implications and future directions

**Unified Trust and Identity Frameworks:** The projects implemented different trust mechanisms (*IDS Connectors, SSI/DIDs, OAuth2, Custom Access Control*). However, a clear need is to research how these mechanisms can be made interoperable or standardised across data spaces. For example, this includes bridging self-sovereign identity models with frameworks like IDS [6] or GAIA-X [7], ensuring scalability and cross-domain trust so that identities and credentials issued in one data space are recognized in another, and establishing well-defined onboarding processes to ensure that participants can trust each other when entering a data space. Moreover, work on governance and certification via state-of-the-art initiatives such as DSSC and Eclipse [98] is needed to harmonize trust services so that data providers and consumers can seamlessly trust each other across data spaces. A complementary step might be the implementation of *micro-certifications* for all involved stakeholders in the implementation process, adopting the idea of *microcredentials* [99]. Additionally, tackling challenges in decentralised policy enforcement and provenance tracking (e.g., improving *blockchain* scalability or *inter-ledger* interoperability) remains an open research area. However, the definition of governance frameworks for European data spaces lies outside the sole scope of individual Horizon Europe projects. This responsibility rests at a higher level, involving the European Commission and the relevant thematic stakeholders of each data space.

**Standardised Metrics for Data Quality and Energy Efficiency:** While many projects introduced metrics for data quality and some for energy, there is a lack of common standards to measure and report these aspects. For instance, agreeing on *a* standard set of energy efficiency indicators for data processing, e.g., by building upon the Green.Dat.AI’s *Energy Efficiency Framework* [34], would help compare and optimise systems across projects. Similarly, a unified approach to data quality benchmarking in data spaces (extending beyond standalone project tools) could enable participants to trust data shared from others based on certified quality scores. However, the absence of standardised energy metrics currently hinders the broader adoption of energy-saving techniques. This means that future work could focus on defining these standards (possibly through bodies like BDVA or DSSC) and developing *tools for automated compliance checking* in live data pipelines [100].

**Scalable and Flexible Business Models:** The creation of business models across the data spaces landscape is still in its early stages. We noticed that the SEDIMARK and Green.Dat.AI projects have introduced concepts like *tokenization* and *reward mechanisms* to encourage data sharing at the pilot level. However, these models should be tested in real-world scenarios to assess their scalability and practical impact. Future research must adopt various ideas, i.e., *marketplaces, data products*, or *bilateral exchanges*, to experiment with what business models actually enable data sharing across various industries. That said, to adequately define business models, it is necessary to identify the governance structures that need to be in place to enable them, i.e., *contracts, pricing structures*, and *revenue-sharing models*. In this context, *regulatory sandboxing* has emerged as a well-used practice in policy development, enabling controlled experimentation with *governance mechanisms* and *compliance tools* before wider adoption. For data spaces in particular, *sandbox environments* provide a practical means to test novel data-sharing models, validate compliance mechanisms under evolving regulatory frameworks, and assess the interoperability of governance tools in real-world settings. For example, the EU AI Act foresees coordinated AI regulatory sandboxes across Member States to foster innovation under regulatory oversight [101]. Similarly, the *OECD Regulatory Sandbox Toolkit* presents sandboxes as learning environments that allow regulators to test new approaches and improve stakeholder engagement [102]. Moreover, recent academic work emphasizes the strategic role of sandboxes and innovation hubs in enabling agile, innovation-friendly regulatory ecosystems [103]. It is also essential to identify how effectively these mechanisms operate in different data sharing contexts*,* e.g., business-to-business (B2B) and business-to-government (B2G) data sharing. *Tools* and *sandbox environments* are also required to enable flexible experimentation with *data or service monetisation strategies* by organizations. *Non-monetary* or *communal benefit models* (data cooperatives, data altruism) may be just as significant as monetized models in industries like agriculture and the public sector, where STELAR and WATERVERSE operate. To ensure that even organizations that are unable to directly profit still have incentives (cost savings, insights, compliance) to contribute data, future research could focus on frameworks that support a variety of sharing business models.

**Advanced Workflow Portability and Automation:** While workflow orchestration is addressed, the integration of *advanced AI technologies* (e.g., *generative AI)* to assist in pipeline design is just beginning (some hints provided in enRichMyData’s AI suggestions for cleaning, or SEDIMARK’s *automated ML pipelines*, or DataBri-X service recommendation engine). Research could enhance the “intelligence” of data space orchestration*,* which includes self-optimising data pipelines that learn from past runs, or virtual assistants that help non-experts build complex workflows using natural language. Moreover, ensuring portability of workflows across different data space platforms is an open challenge: a workflow designed in one project’s toolkit might not easily transfer to another’s. This is reflected in STELAR's perspective to focus on tracking workflow metadata, such as execution parameters and metrics, without imposing any restriction on the specific *workflow engine* being used for execution. Adopting *common workflow description standards* or *containerization practices* should be explored so that data processing recipes can be shared across data spaces (analogous to how *Docker* [104] containers or *Kubernetes* [105] scripts are portable).

**Cross-Domain Data Space Interoperability:** Each reviewed project focuses on an individual domain or use case. As multiple data spaces emerge (health, energy, water, agriculture, etc.), an open question is how they will interconnect or at least avoid forming new silos of formulated data spaces. Further research is warranted on interoperability across data spaces, e.g., mapping *ontologies* from one domain to another, or establishing inter-domain trust mechanisms. The DSSC Blueprint provides a baseline, but real implementations will need to align sector-specific standards so that, for example, a dataset from WATERVERSE could be discovered and used in an agrifood context (e.g., for STELAR) or vice versa when relevant. Thus, an open research challenge is to support higher-level federation of data spaces by creating *cross-data-space catalogues or Connectors* across different domains.

## 5 Summary and conclusion

The value proposition of data management and sharing has grown stronger as the digital transition of Europe gains speed. To unlock the value of data without undermining trust, sovereignty, and innovation, the European Strategy for Data encourages the development of data spaces. Since data spaces are large and complex data ecosystems [106], real-world implementation reveals multiple challenges. The core set of technical and governance requirements necessary to tackle these challenges includes interoperability, workflow orchestration, data quality, discovery, energy efficiency, trust, and sustainable business models. They form a theoretical framework for data management and sharing that borrows ideas from the bibliography.

To test the real-world applicability of this conceptual framework, we examined six representative Horizon Europe projects: *enRichMyData, DataBri-X, Green.Dat.AI, SEDIMARK, STELAR,* and *WATERVERSE*. All projects adhere to common principles such as semantic interoperability, FAIR data management, and federated identity management, though each addresses domain-specific needs. Most of them utilize elements like modular components (e.g., *Connectors*), access control mechanisms, and semantic models that adhere to the DSSC Blueprint [11]. Convergence and divergence have been identified in the projects examined. Regardless of their differing implementations, they all focus on shared standards and practices of governance. *Federated catalogues* and *AI-powered search* are examples of mechanisms of discovery; *blockchain* and *self-sovereign identity* are examples of trust solutions; and business models vary in level of maturity, some with value-added services and others with token-based incentives. The differences highlight the need to create a common technical foundation upon which to tailor data space solutions to particular industries.

The use cases of the examined projects highlight that the social value of data spaces extends much beyond their technological use. They vary from smart manufacturing and smart agriculture to mobility in cities and water management, including aspects such as water quality. All the projects show real accomplishments such as more resilient cities and greener ecosystems, along with increased efficiency across sectors. All of them illustrate the capacity of standardised data ecosystems to create social and economic value. However, some unresolved issues for further research have come to light through our study, indicating there is still a substantial amount of work to be done.

There are still some challenges ahead in terms of standardising the measurement of energy efficiency and data quality, trust frameworks, and inter-data space interoperability. Additionally, for long-term participation by all stakeholders, sustainable business models and governance frameworks must be developed. Nevertheless, the outcomes of these projects create a prosperous foundation for the future European Data Space landscape. They also provide a workable pathway for transforming an open, trusted, and prosperous data sharing environment in Europe through the integration of domain-level innovation with architectural framework principles and ready-to-use tools and services. These endeavours contribute to the development of next-generation data space solutions and aim to fuel the construction of open, large-scale data ecosystems.

Moving forward, the vision is not just to establish stand-alone data space solutions, but to federate them as part of a broader effort to create a pan-European single market for data. The ecosystem should facilitate stakeholders from all sectors with the ability to share, reuse, and monetise data or data-derived services in a secure manner, with backing from trusted governance patterns and a solid value chain. Building on the innovation and expertise from current activities, future research should further investigate how these initiatives can contribute to an open, inclusive, and sustainable European data economy.

While these contributions provide valuable insights, it is also important to acknowledge several limitations of our study. First, the sample of projects that we study is not exhaustive; while the sample we used gives an illustrative picture of the current developments, many other EU-funded projects are also involved in building the landscape of data spaces. Second, the study is qualitative and not hypothesis-driven. Because of heterogeneity across project context and varied maturity levels, we avoided conducting a quantitative study. Instead, we highlighted the search for common trends, new developments, and new emerging gaps, which are all discussed further in Section 4 as potential avenues for future research.

## Funding sources

This work was funded by the HE CSA IDEATION project under the call HORIZON-MISS-2023-OCEAN-01-09 and Grant Agreement No. 101157371. The research leading to the results presented in this paper has received funding from the following European Union-funded projects under the call HORIZON-CL4–2021-DATA-01–03: EnRichMyData under Grant 101070284, DataBri-X under Grant 101070069, Green.Dat.AI under Grant 101070416, SEDIMARK under Grant 101070074 (this project is also partly funded by UK Research and Innovation (UKRI) under the UK government’s Horizon Europe funding guarantee Grant 10043699), STELAR under Grant 101070122, and WATERVERSE under Grant 101070262.

## Declaration of generative AI and AI-assisted technologies in the writing process

During the preparation of this work, the authors used generative AI and AI-assisted technologies (e.g., ChatGPT) solely to improve language clarity and grammar. After using these tools, the authors reviewed and edited the content as needed and took full responsibility for the integrity and accuracy of the final text.

## Data Availability

Horizon Europe SiteGreen.Dat.AI Website (Original data)
Horizon Europe SiteSEDIMARK Website (Original data)
Horizon Europe SiteSTELAR Website (Original data)
Horizon Europe SiteWATERVERSE Website (Original data)
Horizon Europe SiteEnrichmyData Website (Reference data)
Horizon Europe SiteDataBri-X Website (Reference data)

## Appendix Supplementary Materials

Image, application 1
