# Supplementary material for: Leveraging technologies for data management and sharing to foster collaboration and implement data spaces
Source: Data Brief. 2026 Apr 17;66:112769. doi: 10.1016/j.dib.2026.112769 (PMC13158788; doi:10.1016/j.dib.2026.112769)
Supplement: Supplementary file 1 [file mmc1.pdf]

Author contributions: CRediT

Ioannis Chrysakis: Conceptualization, Methodology Project administration, Writing – original draft, Writing – review and editing, Visualization, Supervision

Gerasimos Antzoulatos: Conceptualization, Methodology Project administration, Writing – original draft, Writing – review and editing, Visualization, Supervision

Luis Sánchez: Conceptualization, Methodology, Writing – original draft, Writing – review and editing

Josiane Xavier Parreira: Conceptualization, Methodology, Writing – original draft, Writing – review and editing

Dimitrios Skoutas: Conceptualization, Methodology, Writing – original draft, Writing – review and editing

Iroshani Jayawardene: Conceptualization, Methodology, Writing – original draft, Writing – review and editing

Roberto Di Bernardo: Conceptualization, Methodology, Writing – review and editing, Supervision

Eloisa Vargiu: Conceptualization, Methodology, Writing – review and editing, Supervision

Stefanos Vrochidis: Supervision, Funding acquisition

Erik Mannens: Methodology Project administration, Writing – review and editing, Supervision
